# Enhanced Production of Polymyxin E in* Paenibacillus polymyxa* by Replacement of Glucose by Starch

**DOI:** 10.1155/2018/1934309

**Published:** 2018-10-14

**Authors:** Zhiliang Yu, Zhongqi Sun, Jianhua Yin, Juanping Qiu

**Affiliations:** College of Biotechnology and Bioengineering, Zhejiang University of Technology, Hangzhou 310014, China

## Abstract

Polymyxin E or colistin, produced by* Paenibacillus polymyxa*, is an important antibiotic against Gram-negative pathogens. The objective of this study is to evaluate the effect of starch in fermentation medium on colistin biosynthesis in* P. polymyxa*. The results indicated that replacement of glucose by starch stimulated colistin production and biosynthesis rate. Overall, the stimulation extent was starch concentration-dependent. As expected, addition of starch induced the expression of* amyE* encoding amylase and increased amylase activity in fermentation solution. Additionally, replacement of glucose by starch resulted in residue reducing sugar and pH of fermentation mixture low relative to glucose as the sole sugar source. At the molecular level, it was found that replacement of glucose by starch has enhanced the relative expression level of* ccpA* encoding catabolite control protein A. Therefore, the repression of starch utilization by glucose could be probably relieved. In addition, use of starch stimulated the expression of regulatory gene* spo0A* but repressed the expression of another regulatory gene* abrB*. As a result, the expression of genes directly involved in colistin biosynthesis and secretion increased, indicating that at the transcriptional level* spo0A* and* abrB* played opposite roles in regulating colistin biosynthesis in* P. polymyxa*. Taken together, our data demonstrated that starch instead of glucose can promote colistin production probably by affecting the expression of colistin biosynthesis-related genes, as well as reducing the repression of glucose to a secondary metabolic product.

## 1. Introduction

Polymyxin E, also called colistin, is an important old antibiotic known for around six decades for treatment of infection caused by Gram-negative pathogens [1, 2]. Later studies showed that colistin can also kill Gram-positive bacteria [3, 4]. Currently, its clinical use is broadly restricted regarding its toxicity mainly to the kidney and nervous system [5]. In recent, the occurrence of Gram-negative multidrug-resistant pathogens which are resistant to many available antibiotics has revived its clinical application in healthcare centers, since colistin is broadly considered as one of the last-line options of antibiotic therapy for multidrug-resistant bacteria. Therefore, its market demand is increasing [6, 7].

Colistin is composed of two parts: a cyclic heptapeptide and a tripeptide side chain which is acylated by a fatty acid at the amino terminus [8, 9]. In total, colistin has ten orderly assembled amino acid residues [10]. Among them, six are L-2,4-diaminobutyric acids (L-Dabs). L-Dab is biosynthesized by 2,4-diaminobutyrate aminotransferase (EctB) which is encoded by* ectB* [11]. Colistin can be biosynthesized by a multienzyme nonribosomal peptide synthetase system (NRPS) in* Paenibacillus polymyxa* [3, 4]. The phosphopantetheinyl transferase (Sfp) encoded by* sfp* is important for colistin biosynthesis due to its activation function on NRPS [12, 13]. A gene cluster including five open reading frames,* pmxA*,* pmxB*,* pmxE*,* pmxC*, and* pmxD*, encoding three synthetases PmxA, PmxB, and PmxE, and two membrane transporters PmxC and PmxD, respectively, has been characterized for biosynthesis and secretion of colistin in* P. polymyxa* [10, 14]. It has been determined that colistin biosynthesis is negatively regulated by AbrB, a DNA-binding protein, by directly binding to the upstream region of* pmxA *[11]. The expression of* abrB* itself is negatively controlled by Spo0A, another DNA-binding protein, encoded by* spo0A* [15]. These two genes play opposite roles in regulation of colistin production.

So far, colistin has been best characterized with respect to its structure and biosynthesis, antibacterial mechanism and bacterial resistance, and toxicity and derivatives. In contrast, extremely little is known about medium optimization for its fermentation output. It has been reported that colistin production depends on the inorganic phosphate concentration [16]. Further optimization showed that addition of L-Dab as well as its precursor aspartic acid to fermentation medium containing appropriate PO_4_^3−^ stimulates colistin production [16]. However, other studies showed that addition of either L-Dab or aspartic acid to medium after 35 h fermentation significantly inhibits colistin production by suppressing the expression of* pmxA* and* pmxE*, as well as* ectB *in another producer strain [17]. Although it has been found that corn meal in the medium is essential for the better production of colistin [16], glucose is the most widely used carbon source for colistin biosynthesis [18]. In our previous study, glucose was also used as a sugar source for colistin production in* P. polymyxa* C12 and its production reached around 6.2 × 10^4^ U/mL (2600 *μ*g/mL) in flask level [17]. As a concern, glucose has been widely found to repress the accumulation of secondary metabolic compounds in microbes through carbon catabolite repression (CCR) [19]. CCR in microbes is regarded as the mechanism in which bacteria preferentially utilize the rapidly metabolizable carbon source (normally glucose). As a result, the utilization of secondary carbon resource is repressed. CCR is considered to be a part of the global control system and therefore it affects many genes [20]. In Gram-positive bacteria, the catabolite control protein A (CcpA) is the master regulator of CCR. Various physiological processes in Gram-positive bacteria are regulated by CcpA [21–23].

In this study, we attempt to substitute starch for glucose and investigate the effect of the sugar source on colistin production in* P. polymyxa* C12. In addition, the effect of the sugar source on amylase activity and relative expression of genes associated with colistin biosynthesis was also evaluated.

## 2. Materials and Methods

### 2.1. Strain and Culture Conditions

Colistin-producer* P. polymyxa* C12 [17] used in this study was frozen at −80°C in our lab at Zhejiang University of Technology, China. Unless otherwise stated,* P. polymyxa* was firstly cultivated on a culture medium agar plate (10 g/L of beef extract, 15 g/L of peptone, 10 g/L of glucose, 2 g/L of yeast extract, 3 g/L of NaCl, 0.1 g/L of FeSO_4_·7H_2_O, and 20 g/L of agar, pH 7.0) at 30°C for 2 d. Then, a ring of* P. polymyxa* was transferred to 50 mL of seed medium (30 g/L of soybean meal, 5 g/L of soybean oil, 0.1 g/L of FeSO_4_·7H_2_O, 15 g/L (NH_4_)_2_SO_4_, 0.77 g/L of KH_2_PO_4_, 0.7 g/L of CaCO_3_, and 10 g/L of glucose, pH 7.0) in a 250 mL flask for incubation at 30°C for 24 h with a shaking at 200 rpm. Next, 5 mL of cell culture was transferred to 50 mL of fermentation medium (23.9 g/L of soybean meal powder (Zhejiang Qianjiang Biochemical Co., Ltd., China), 21.1 g/L of soybean cake powder (Zhejiang Qianjiang Biochemical Co., Ltd., China), 10 g/L of soybean oil, 0.1 g/L of FeSO_4_·7H_2_O, 25 g/L (NH_4_)_2_SO_4_, 0.77 g/L of KH_2_PO_4_, 1 g/L of CaCO_3_, and 45 g/L of glucose, pH 7.0) in a 250 mL flask at 30°C with shaking at 200 rpm for fermentation. A certain amount of glucose from 20 g/L to 45 g/L in the fermentation medium was replaced by starch if necessary. Unless otherwise specified,* P. polymyxa* was fermented for 96 h and 0.5 mL of samples was collected per 12 h. The colistin concentration and relevant gene expression were determined by HPLC and quantitative real-time PCR (qRT-PCR), respectively.

### 2.2. Measurement of Cell Growth

Unless otherwise stated, the bacterial biomass of the cultured cells was determined based on the value of colony forming unit (CFU) [24, 25]. First, the cells were collected after centrifugation at 5,000 g for 5 min. After washing twice with 0.5 mL of fresh broth culture medium (10 g/L of beef extract, 15 g/L of peptone, 10 g/L of glucose, 2 g/L of yeast extract, 3 g/L of NaCl, and 0.1 g/L of FeSO_4_·7H_2_O, pH 7.0), the cells were then resuspended in 0.5 mL of fresh broth culture medium. Then, the cells were tenfold gradiently diluted. Finally, 100 *μ*L of cells was spread to a culture medium agar plate for growth. After cultivation at 30°C for 2 d, CFU were counted.

### 2.3. HPLC Analysis of Colistin

One milliliter of fermentation liquor and 9 mL of ultrapure water were mixed. Then, 1 mL of diluted fermentation liquor was centrifuged at 10,000 g for 10 min and the supernatant was collected and filtered with 0.45 *μ*m hydrophilic microporous membrane (Millipore). Analysis of colistin was performed using binary gradient model of an HPLC system (SHIMADZU, Japan). Twenty *μ*L of supernatant sample was injected into a reverse-phase column, YMC Pack ODS-A (150 × 4.6 mm I.D., 5 *μ*m), eluted at 33°C, and analyzed in a mixed solvent of acetonitrile (22%) and water containing 0.223% Na_2_SO_4_ (78%), at a constant flow of 1 mL/min. The mixed solvent was prepared by mixing pure acetonitrile in A-pump with water containing 0.223% Na_2_SO_4_ in B-pump. Separation program was set as follows: 0~20 min, 22% A-pump and 78% B-pump; 21~30 min, 90% A-pump and 10% B-pump; 31~40 min, 22% A-pump and 78% B-pump. Colistin peak was determined at wavelength of 240 nm. Colistin concentration produced was calculated based on the extracted correlation between the concentration of standard colistin (Zhejiang Qianjiang Biochemical Co., Ltd., China) and the corresponding peak area in HPLC. One unit is equal to 0.0418 *μ*g of colistin [17]. Colistin was dissolved in 1 mL of pure water to make 2 × 10^5^ U/mL solution. Then, colistin was fivefold gradiently diluted with water to make serial colistin solutions. For HPLC analysis, 20 *μ*L of standard colistin was injected.

### 2.4. Detection of Reducing Sugar and Assay of *α*-Amylase Activity

After fermentation, the cell mixture was centrifuged at 10,000 g for 10 min and the supernatant was collected. Unless otherwise specified, the amylase activity in the fermentation supernatant (crude enzyme solution) was determined by measuring the reducing sugar generated during the reaction [26]. In general, 0.2 mL of 0.1 M citrate-phosphate buffer with pH 7.0 containing 1% (w/v) soluble starch was preheated at 30°C for 5 min. Then, 0.05 mL of crude enzyme solution appropriately diluted with sterilized ultrapure water was added and mixed thoroughly. After incubation at 30°C for 30 min, the reaction was terminated by adding 1 mL of DNS reagent containing 182 g/L Rochelle salt, 21 g/L NaOH, 6.3 g/L dinitrosalicylic acid, 5 g/L crystalline phenol, and 5 g/L Na_2_SO_3_. Next, the mixture was placed in boiling water for 5 min, followed by cooling down to room temperature. Finally, the absorbance value of mixture at 540 nm was measured [26]. The concentration of reducing sugar was determined based on the extracted correlation between standard glucose over a range of concentrations and the corresponding absorbance values at 540 nm. One unit of amylase activity was defined as the amount of enzyme required to release 1 *μ*g of glucose equivalent per minute under the assay condition using glucose as the standard.

### 2.5. PCR Amplification and Sequence Retrieval of Genes

The sequences of* pmxABCDE*,* spo0A*,* abrB*,* ectB*, and* sfp* have been amplified and collected in our previous study [17]. In this study, the primers (Table 1) for* amyE* encoding amylase and* ccpA* encoding CcpA in PCR reaction were designed based on the complete genome sequence of* Paenibacillus polymyxa* SC2 (GenBank access no. CP002213.2). Bacterial genomic DNA was extracted using a bacterial genomic DNA extraction kit (GE, USA). PCR reaction was performed as reported previously [27, 28]. In brief, the gene fragments were amplified in 50 *μ*L containing 37 *μ*L of ddH_2_O, 5 *μ*L of 10X EasyTaq buffer, 4 *μ*L of 2.5 mM dNTPs, 100 nM forward primer, 100 nM reverse primer, 1 ng genomic DNA, and 1 U* Taq* DNA polymerase (TaKaRa, Dalian, China) with denaturation at 94°C for 5 min followed by 30 cycles of 1 min at 94°C, 50 s at 55°C, 90 s at 72°C, and a final 10 min extension at 72°C. At the end of reaction, PCR product was cooled to 4°C for further use. After size confirmation on 1.0% agarose gel, the desired amplicons were purified using a gel extraction kit (Qiagen, CA, USA) for TA cloning with pMD19-T simple vector (TaKaRa, Dalian, China). After sequencing by Sangon Biotech (Shanghai, China), the gene sequences were collected and compared with the reference genes in GenBank for confirmation.

### 2.6. qPCR Analysis of Gene Expression

In brief, 0.5 mL of the bacterial cells was pelleted after centrifugation at 8,000 rpm for 10 min at 4°C and the total RNA was extracted using an RNAiso Plus kit (TaKaRa, Dalian, China) according to the manufacturer's instructions. RNA purity was spectrophotometrically evaluated based on OD_260 nm_/OD_280 nm_ ratio. Then, 300 ng of DNA-free RNA was reversely transcribed to cDNA in a 10 *μ*L volume using PrimeScript™ RT Master Mix (Perfect Real Time) kit (Toyobo, Tokyo, Japan). After appropriate dilution, the obtained cDNA was used for amplification of target gene fragment with primer sets (Table 2) [17] by using the SYBR green* Premix Ex Taq*™ (Tli RNaseH Plus) kit. A master mixture was prepared and each well of reaction contained the following reagents: 5 *μ*L of SYBR Green Master Mix, 0.2 *μ*L forward primer and reverse primer, respectively, and 3.6 *μ*L of ddH_2_O to a total of 9 *μ*L. After addition of 1 *μ*L of each diluted cDNA sample to each well, the PCR was run on CFX Connect Real-Time System (Bio-Rad, Hercules, CA) with an amplification protocol consisting of an initial denaturation at 95°C for 10 min, followed by 40 cycles of denaturation at 95°C for 15 s and annealing/elongation at 60°C for 30 s. Immediately after the final cycle of PCR, melting curve was analyzed to retrieve the specificity of the reaction based on the observation of melting temperature from the product [29].

The cycle threshold (*C*_T_) for each PCR was determined using StatView software which automatically set the threshold signal at the log phase of amplification curve. The amplification efficiency of gene was retrieved from the slope of that linear regression according to the formula* E*=10^(-1/slope)^. Several dilutions of each cDNA sample were assayed for the gene of interest in order to obtain a linear regression between the *C*_*T*_ values (ranging from 15 to 35 cycles) and the log of cDNA. The 116 bp of 16S rRNA gene fragment ranging from 16SF (5′-GAGAAGAAAGCCCCGGCTAA-3′) to 16SR (5′-ACCAGACTTAAAGAGCCGCC-3′) was used as the internal control to verify that there was an equal amount of target cDNA in all samples. The expression of target gene relative to 16S rRNA gene was calculated as described in report [30].

### 2.7. Data Analysis and Availability

Unless otherwise specified, triplicate reactions per experiment were performed. All data were presented as mean ± standard error and tested for statistical significance based on analysis of variance (ANOVA) followed by Dunnett's post hoc test using StatView 5.0 program. When the probability (p) was less than 0.05 and 0.01, the values were considered significantly (*∗*) and very significantly (*∗∗*) different, respectively.

## 3. Results

### 3.1. Dependence of Starch Concentration on Colistin Biosynthesis

**Figure 1:**
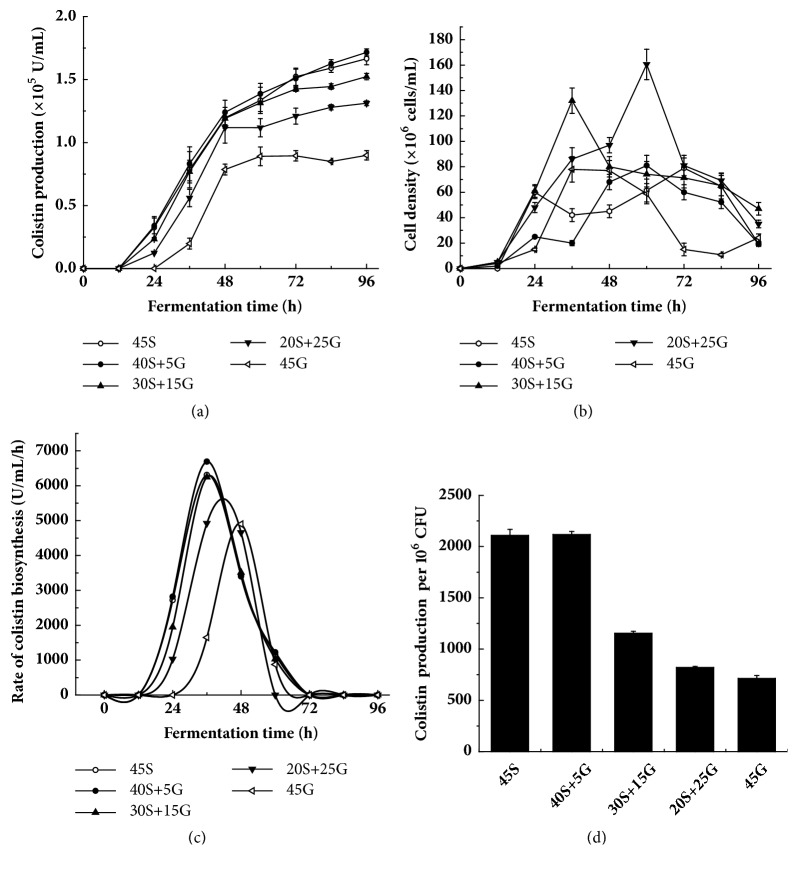
Influence of replacement of glucose by starch on colistin production in* P. polymyxa*. (a) Colistin production along fermentation; (b) growth curve of* P. polymyxa* along fermentation; (c) rate of colistin biosynthesis along fermentation; (d) colistin production per cell biomass after 96 h fermentation. 45S: 45 g/L starch; 40S+5G: 40 g/L starch plus 5 g/L glucose; 30S+15G: 30 g/L starch plus 15 g/L glucose; 20S+25G: 20 g/L starch plus 25 g/L glucose; 45G: 45 g/L glucose.

To investigate the effect of starch on colistin accumulation in* P. polymyxa* C12, different amounts of glucose in fermentation medium were replaced by starch. As shown in Figure 1(a), colistin was undetectable within the first 24 h using 45 g/L glucose as the sole sugar source. Then, its production rapidly increased up to 48 h, followed by almost a constant in the remaining period. The highest yield of colistin was around 8.5 × 10^4^ U/mL. Instead, the replacement of glucose by starch in fermentation medium gave detectable colistin at 12 h. Next, colistin production rapidly increased also up to 48 h, followed by a moderate increase in the remaining period. Overall, the more the glucose was replaced by starch, the higher the colistin was produced. The highest yield of colistin with 40 g/L starch plus 5 g/L glucose was around 1.66 ×10^5^ U/mL, approximately one time higher than the one with 45 g/L glucose. The 45 g/L starch as the sole sugar source showed a similar result to 40 g/L starch plus 5 g/L glucose.  Figure 1(b) indicated that the proportion of sugar source clearly affects the cell growth. Overall, the high proportion of glucose was beneficial to cell accumulation for early stage of fermentation. In contrast, the high proportion of starch was beneficial to cell accumulation for later stage of fermentation. Most probably, the use of glucose is faster than that of starch.  Figure 1(c) showed that the rate of colistin biosynthesis with 45 g/L glucose as the sole sugar source rapidly increased and then decreased. The highest rate of colistin biosynthesis was 4.8 × 10^3^ U/(mL·h) at 48 h. Similarly, the rate of colistin biosynthesis with all mixtures of glucose and starch rapidly increased and then decreased. Interestingly, the highest rate of colistin biosynthesis overall appeared earlier and higher with the increase of replacement of glucose with starch. The highest rate of colistin biosynthesis with 40 g/L starch plus 5 g/L glucose reached 6.7 × 10^3^ U/(mL·h) at 36 h.  Figure 1(d) further showed that the replacement of glucose with starch enhanced the colistin production per biomass. Overall, the more the glucose was replaced, the higher the colistin per biomass was produced. The highest yield of colistin per biomass with 40 g/L starch plus 5 g/L glucose was around 2.1 × 10^3^ U/(mL·10^6^ CFU), approximately two times higher than the one with 45 g/L glucose. The 45 g/L starch as the sole sugar source displayed a similar result to 40 g/L starch plus 5 g/L glucose. All these data congruously indicated that the replacement of glucose with starch stimulates the colistin accumulation in* P. polymyxa*.

### 3.2. Effect of Starch on Amylase Activity and Relative Expression of amyE

**Figure 2:**
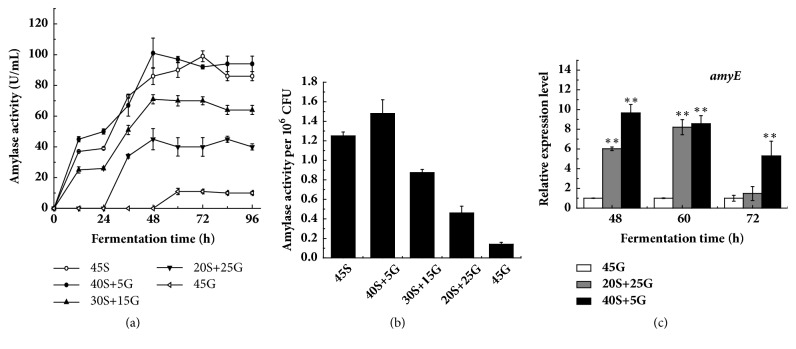
Influence of replacement of glucose by starch on amylase and gene expression. (a) Amylase activity along fermentation; (b) amylase activity per cell biomass after 96 h fermentation; (c) relative expression level of* amyE*. 45S: 45 g/L starch; 40S+5G: 40 g/L starch plus 5 g/L glucose; 30S+15G: 30 g/L starch plus 15 g/L glucose; 20S+25G: 20 g/L starch plus 25 g/L glucose; 45G: 45 g/L glucose. The statistically significant results are related to the condition of 45 g/L glucose.

Starch should be decomposed by amylase before use in fermentation. Therefore, amylase activity was monitored. As shown in Figure 2(a), amylase activity with 45 g/L glucose as the sole sugar source can be detected at 48 h. Then, it increased to 11 U/mL at 60 h, followed by almost a constant in the remaining period. Amylase activities with both 40 g/L starch plus 5 g/L glucose and 45 g/L starch were around 100 U/mL at 48 h and 72 h, respectively, eight times higher than the one with 45 g/L glucose.  Figure 2(b) further showed that amylase activity per 10^6^ CFU with 40 g/L starch plus 5 g/L glucose was around 1.48 U/mL, eight times higher than 0.17 U/mL of amylase activity per 10^6^ CFU with 45 g/L glucose.  Figure 2(c) indicated that the relative expression level of* amyE* encoding amylase significantly increased with increase of starch replaced for glucose. All these results supported the reports that the transcription of* amyE* is strongly increased by starch [31] but repressed by glucose [32].

### 3.3. Effect of Starch on pH and Reducing Sugar Formation

**Figure 3:**
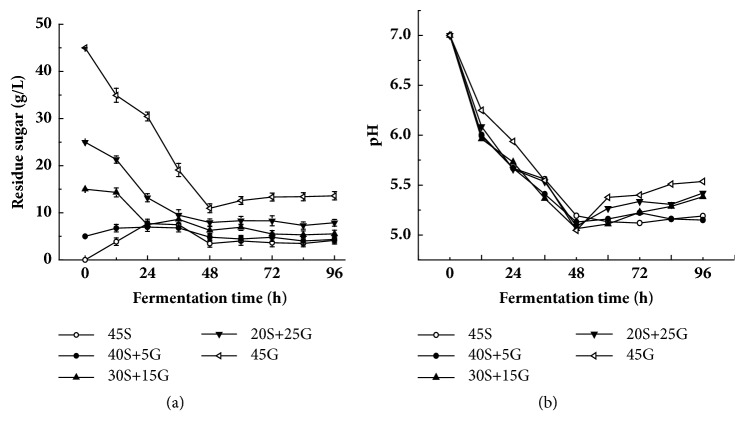
Influence of replacement of glucose by starch on residue reducing sugar (a) and pH (b) during fermentation. 45S: 45 g/L starch; 40S+5G: 40 g/L starch plus 5 g/L glucose; 30S+15G: 30 g/L starch plus 15 g/L glucose; 20S+25G: 20 g/L starch plus 25 g/L glucose; 45G: 45 g/L glucose. The statistically significant results are related to the condition of 45 g/L glucose.

Carbon source could affect reducing sugar and accordingly fermentation output [33, 34].  Figure 3(a) showed that the residue reducing sugars in fermentation with original glucose ranging from 15 g/L to 45 g/L decreased within the first 48 h and kept almost constant in the second 48 h. Most probably, the consumption of glucose in fermentation medium could result in the decrease of reducing sugar within the first 48 h. The higher the original concentration of glucose in fermentation medium was, the faster the reducing sugar decreased within the first 48 h. On the contrary, the residue reducing sugar in fermentation with original glucose ≤15 g/L increased within the first 48 h and kept almost constant in the second 48 h. Most probably, the rate of decomposition of starch in fermentation medium would surpass the rate of reducing sugar consumption within the first 48 h, thus resulting in the increase of reducing sugar.  Figure 3(a) further indicated that the higher the original concentration of glucose was, the higher the concentration of residue reducing sugar was at the end of fermentation. The residue reducing sugars in fermentation medium with 45 g/L glucose and 45 g/L starch as original sugar were 13.6 g/L and 4.1 g/L, respectively, at 96 h.  Figure 3(b) showed that the pH of fermentation solution with different sugar sources displayed a similar pattern, rapid decrease within the first 48 h and slight increase within the second 48 h. Overall, the higher the original glucose concentration in fermentation medium was, the higher the pH of fermentation solution was within the second 48 h, which is negatively correlated with colistin production (Figure 1). The fact that the overall difference in pH of fermentation solution derived from proportion of sugar source is visible, but not remarkable, is worth noting.

### 3.4. Effect of Starch on Relative Expression of Genes for Regulation of Colistin Biosynthesis

**Figure 4:**
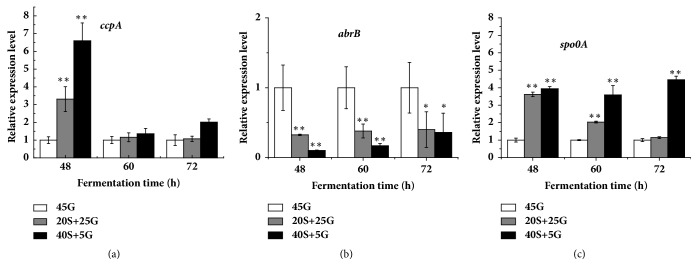
Effect of replacement of glucose by starch on relative expression level of genes involved in regulation of colistin biosynthesis. (a)* ccpA*; (b)* abrB*; (c)* spo0A*. 45G: 45 g/L glucose; 20S+25G: 20 g/L starch plus 25 g/L glucose; 40S+5G: 40 g/L starch plus 5 g/L glucose. The statistically significant results are related to the condition of 45 g/L glucose.

CcpA encoded by* ccpA* is the master regulator of CCR in Gram-positive bacteria and it can affect the expression of the* abrB* [35]. Both* abrB* and* spo0A* are believed to be associated with colistin production [17]. Therefore, the relative expression of these three genes was investigated.  Figure 4 showed that* ccpA* and* spo0A* gave overall similar patterns in relative gene expression. The higher the original concentration of glucose in fermentation medium was, the lower the relative expression of either* ccpA* or* spo0A* was. Interestingly,* abrB* had the opposite pattern in relative gene expression. The higher the original concentration of glucose in fermentation medium was, the higher the relative expression of* abrB* was. All these results indicated that the replacement of glucose by starch can stimulate the expression of both* ccpA* and* spo0A* but repress the expression of* abrB*, which in turn stimulates colistin production (Figure 1).

### 3.5. Effect of Replacement of Glucose by Starch on the Relative Expression of Genes Directly Involved in Colistin Biosynthesis

**Figure 5:**
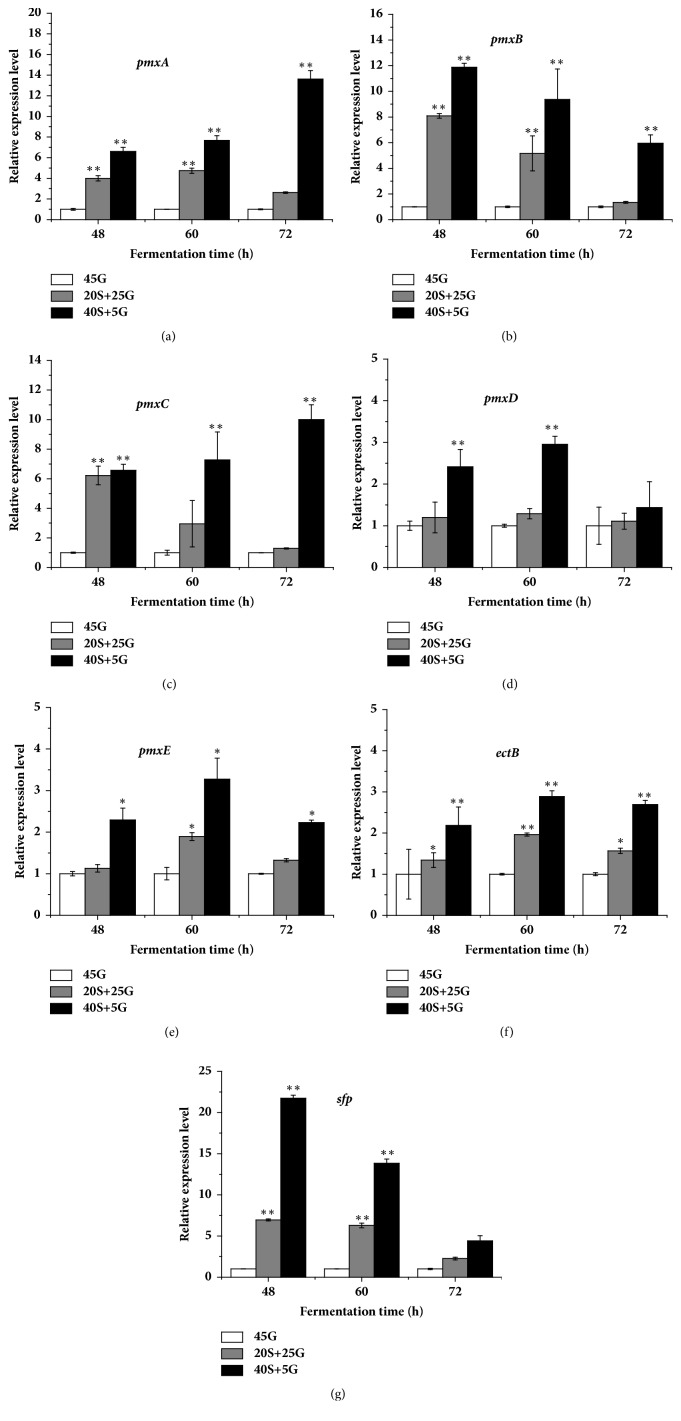
Effect of replacement of glucose by starch on relative expression level of genes directly involved in colistin biosynthesis and secretion. (a)* pmxA*; (b)* pmxB*; (c)* pmxC*; (d)* pmxD*; (e)* pmxE*; (f)* ectB*; (g)* sfp*. 45G: 45 g/L glucose; 20S+25G: 20 g/L starch plus 25 g/L glucose; 40S+5G: 40 g/L starch plus 5 g/L glucose. The statistically significant results are related to the condition of 45 g/L glucose.

As shown above, the replacement of glucose by starch can promote the relative expression of genes associated with regulation of colistin production. Therefore, the relative expression levels of genes directly involved in colistin biosynthesis and secretion were examined.  Figure 5 showed that the replacement of glucose by starch stimulated the relative expressions of* pmxABCDE*,* ectB*, and* sfp*. The more the glucose was replaced, the higher the relative expressions of those genes were, indicating that the replacement of glucose by starch can promote the expression of those genes and in turn increased colistin production (Figure 1).

## 4. Discussion

Colistin is broadly used to treat the infection of Gram-negative pathogens, particularly prevalent multidrug-resistant bacteria. It is produced by* P. polymyxa*. To date, very few reports dealt with the medium optimization for improvement of colistin production. In the present study, the effect of replacement of glucose by starch in fermentation medium on colistin production as well as transcription level of colistin biosynthesis-related genes was investigated. It was found that addition of starch could improve the production and biosynthesis rate of colistin (Figure 1). Moreover, the improvement extent was positively correlated with the amount of glucose replaced by starch (Figure 1). Our data further showed that the replacement of glucose by starch could refine two important fermentation factors, residue reducing sugar and pH (Figure 3). It seems that low concentration of residue reducing sugar and pH is better for colistin biosynthesis in* P. polymyxa* (Figure 1), but the detailed correlation mechanism needs to be further explored.

It has been found that the use of glucose represses the amylase activity and sporulation [36]. A report has shown that, relative to other carbon sources, glucose causes the strongest CCR, reducing the production of secondary metabolite [37]. It has been revealed that CCR is achieved by the global transcription regulator CcpA. The expression of* ccpA* results in the reduction of CCR [22]. Therefore, CcpA positively regulates secondary metabolism [23]. Our results showed that the replacement of glucose by starch could increase the relative expression of* ccpA* (Figure 4). As a result, the replacement of glucose by starch increased the transcription of* amyE* and amylase activity (Figure 2), which in turn is probably conducive to colistin (secondary metabolite) accumulation in* P. polymyxa* (Figure 1). Therefore, our findings are in line with the reports [22, 23, 36].

It has been demonstrated that Spo0A positively regulates secondary metabolism [38]. Our results indicated that the use of starch enhanced the relative expression level of* spo0A* (Figure 4) and subsequently increased colistin production (Figure 1), suggesting that* spo0A* also positively affects colistin biosynthesis in* P. polymyxa* at the transcriptional level. Therefore, our findings are in line with the report [38]. It has been found that the expression of* abrB* is negatively regulated by Spo0A [15]. Our results also indicated that the relative expression level of* abrB* decreased with the increase of* spo0A* expression (Figure 4). There is evidence to show that AbrB negatively regulates colistin biosynthesis by directly binding to the upstream region of* pmxA *[11]. Thus, the decrease of* abrB* expression by adding starch (Figure 4) enhanced the relative expression of* pmxABCDE*, a gene cluster for colistin biosynthesis (Figure 5). As a result, colistin accumulation increased (Figure 1).

## Figures and Tables

**Table 1:** Sequences of primer pairs for PCR amplification of genes.

Genes	Nucleotide sequences (5′-3′)		Product sizes (bp)
	Forward primers	Reverse primers	
*amyE*	ATGGTCCACAATCCTGTT	CCTCATGTTCTTCCCTCA	1319
*ccpA*	TGGTCAGCAAACGCATCG	AAACCTCAGACCCGCAAG	832

**Table 2:** Sequences of primer pairs for real-time PCR analysis of gene expression.

Genes	Nucleotide sequences (5′-3′)		Product sizes (bp)
	Forward primers	Reverse primers	
*pmxA*	TCAACTCGCTCAGAAGCGTT	TTGTACGGAAACCGACGGAG	105
*pmxB*	ATGAAATCTTTGTTTGAAAA	CCAGGACGTACACCCTCAAC	111
*pmxC*	TATTCCCGAGCTCATCACGC	TCGGAAGCGAACGACCATTT	107
*pmxD*	TGTTCGTTCAACGCCTCGTA	GCTTGCAAACGCTCGGTAAA	118
*pmxE*	CACTTTGCCTGAAACGACCG	GCCAGAATGCGTTCATACCG	111
*spo0A*	TCGCAGAATCCCGCAACATA	CGGTTGTGGAGTCAGGTTCA	103
*abrB*	AAATACGGAACAGCCCGTCC	TCGCTCGCCTGTCTTCAAAT	114
*ectB*	CAGTGGATACGGTCTGCCAA	CTCCGACAAACGCTAGCTGA	113
*sfp*	GTACCTCCTGCGCAAAGTGA	CACGACAGAGGGCTTTACGA	110
*amyE*	TCTGGGCGGAACGATTTTGA	CGAGTGCCGCCCTATTGTAT	110
*ccpA*	ATCAATTCCGGCTGCTTCCA	CACCGCCAAATCGCAATGAT	102

## Data Availability

All the data used to support the findings of this study are available from the corresponding author upon request.
